# Timing Matters: Leveraging Temporal Contexts for Interpreting Reading Progress

**DOI:** 10.1111/mbe.70020

**Published:** 2025-10-24

**Authors:** Joanna A. Christodoulou, Laura Mesite, J. Adam Hickey

**Affiliations:** 1 Department of Communication Sciences and Disorders, MGH Institute of Health Professions; 2 Harvard Graduate School of Education; 3 Landmark School

## Abstract

Improving reading outcomes for students with reading disabilities/difficulties (RD) has been commonly studied relative to ‘what’ a reading intervention entails. Here, we offer a broader perspective on ‘when’ we implement reading services to improve our interpretation of reading progress. We address several aspects related to timeframes (i.e., temporal contexts) to contextualize reading changes associated with score increase or decrease trends. First, we review learning trajectories that can provide baseline expectations for reading and changes among students with RD. Second, we call attention to fade-out effects, which describe the positive impact of intervention programs gradually neutralizing in the years following program completion. Third, we discuss learning outcomes associated with suspended formal schooling, including the expected summer vacation from school and the unexpected COVID-19 school interruptions. We highlight the value of contextualizing reading progress in broader circumstances and conditions for readers.

Understanding and interpreting changes in reading performance is a complex task, particularly when considering students with reading difficulties or disabilities (RDs). While much attention has been given to the content and methods of reading interventions, less focus has been placed on the role of timing—when and for how long reading interventions are delivered (or suspended) relative to the other conditions in a reader’s literacy experiences. Temporal contexts, or the timing and duration of educational experiences, play a critical role in shaping reading development. This article highlights the importance of contextualizing reading score changes within these time-based frameworks to better evaluate progress, identify challenges, and design effective support strategies.

Temporal contexts can shape when and how reading skills are acquired, practiced, and retained, or conversely, the consequences of paused instruction, waning of literacy practice, or skill attrition. Skill learning is most readily interpretable through a *dynamic skills framework*, which describes a flexible learning system that balances skill progression and regression, responsive to available instruction, support, and experience (De Bot, Lowie, & Verspoor, 2007; Fischer & Bidell, 2006). This theory lends a systematic expectation for reading performance to vary depending on educational conditions, with potential attrition during prolonged insufficient opportunities or resources, and skill advancement under supportive and conducive conditions. Thus, interpreting reading intervention score changes in broader contexts of literacy experiences can improve our precision in attributing drivers of reading progress.

Temporal contexts can also provide insights into whether changes might be expected or unexpected. For example, reading score declines might be anticipated during prolonged school closures (e.g., during the COVID-19 pandemic) (NAEP, 2008) but would be unexpected following an intervention program meant to improve reading skills (Suggate, 2016). Without considering these temporal factors, educators risk misattributing performance shifts. Further-more, exploring temporal contexts specific to students with RD can help clarify trends that are shared or distinct from non-RD peers.

We highlight temporal contexts important for interpreting changes in reading performance, particularly for students with RD, whose reading acquisition often requires consistent and carefully timed support. While much of the research on RD focuses on the content and methods of interventions, our discussion emphasizes the importance of timing—*when* services are delivered or retracted—as a critical factor in understanding reading progress. Thus, we move beyond the typical emphasis on available literacy resources to address the consequences of their absence and the disruptions this can cause to anticipated progress or skill maintenance.

We explore three key types of temporal contexts: learning trajectories, fade-out effects, and interrupted schooling. Learning trajectories provide baseline expectations for reading skill development, allowing educators to determine whether progress aligns with expected patterns. While many studies of reading trajectories evaluate broad populations of readers, we focus on trajectories specific to students with RD. Next, we focus on fade-out effects, which underscore how the benefits of intervention programs can diminish over time without sustained reinforcement. Given the high-stakes and time-sensitive needs of students with RD, considering long-term sequelae of interventions and continuity of services can aim to avoid anticipated fade-out effects. We also examine the learning outcomes associated with interruptions in formal schooling, including the summer slide and COVID-19 pandemic-related disruptions. By contextualizing reading progress within these time-based frameworks, educators and clinicians can make more informed decisions, design more impactful interventions, and better support students with RD to optimize long-term and sustained literacy progress.

## READING SKILL TRAJECTORIES IN RD

The trajectories of students’ reading skills, especially for those with identified RDs, can provide valuable insights into individual differences, typical rates of change, and predictors of outcomes. To address this topic, we highlight the trajectories specific to US students with reading disabilities identified primarily in elementary school (Connors et al., 2025; Ferrer et al., 2015; Ferrer, Shaywitz, Holahan, & Shaywitz, 2023; Shaywitz et al., 1999) and report a case study of longitudinal reading scores among students with RD to expand on trajectories often discussed for broader groups of students (i.e., predominantly non-struggling readers, at-risk readers, or students with broader learning challenges). Longitudinal studies of reading focusing on students with identified reading disabilities can help interpret intervention outcomes through expectations for common growth trajectories as well as expected variation by skill level, grade, time period, and individual differences. These data can also guide educational planning and intervention design, with the broader goal of reducing the performance gap between students with and without RD.

Learning trajectory information often includes several key aspects of reading performance. These aspects may include scores at school entry (e.g., at kindergarten), the pace of change (e.g., increase, maintenance, or decline), the shape of change (e.g., linear and exponential), variability in change, and how demographic or contextual factors (e.g., reading disability status) influence these indices. Though less commonly reported, studies examining reading trajectories could be informative by specifying reading curricula, dosage, and implementation quality; teacher knowledge, skills, and effectiveness; reading experiences outside of formal instruction; and dynamic or static neuropsychological aspects (e.g., attention, executive function, cognition; motivation, self-concept, and engagement).

Among studies focused on reading trajectories for participants with reading disabilities, converging findings point to differences present at least as early as elementary school that persist into adulthood. For example, Connors et al. (2025) identified lower reading scores for students with versus without reading/math disabilities in grade 1. The score gaps continued, as indicated by parallel reading trajectories for public school children with reading disabilities (*n* = 74) versus children with no reading (or math) disabilities (*n* = 270) from grades 1 (fall of 2015/2016) to grade 5 with similar shapes and patterns of initial improvement and gradual slowing of progress over time. This study also accounted for prior reading intervention, reporting gains in decoding and oral reading fluency (as well as math fluency) among recipients, both relative to their own pre-intervention performance and to peers who did not receive the intervention (potential fade-out effects were not addressed).

Another set of analyses from the Connecticut Longitudinal Study (CLS) followed students entering public kindergarten (1983–1984 school year), including those identified with dyslexia in elementary school (Ferrer et al., 2015, 2023; Shaywitz et al., 1999). In addition to identifying largely parallel reading trajectories that show initial accelerated growth followed by a slower pace of change, Shaywitz et al. (1999) found that reading scores over time were consistently highest for superior readers (*n* = 39), intermediate for average readers (*n* = 35), and lowest for the persistent RD group (*n* = 21), who did not show evidence of closing gaps over time. Survey data related to experiences in and out-of-school settings, collected in grade 12, revealed some group differences, including students with dyslexia being less likely than their peers to borrow library books or spend time reading.

Also drawing on the CLS, Ferrer et al. (2015) reported that students with dyslexia (*n* = 79) performed below typically reading peers (*n* = 335) in first grade on reading-related measures. These reading score gaps largely persisted when measured again at grade 12, even when rates of progress were slightly faster for readers with dyslexia compared to typical readers (on word identification and passage comprehension). Their extension of this study into adulthood found performance gaps remained between dyslexia (*n* = 66) and typical reader (*n* = 246) groups at age 42. While reading scores in grade 1 predicted adult comprehension similarly for both dyslexia and typical reader groups, the pattern of reading growth from first to fifth grade was a stronger predictor of adult reading comprehension outcomes for those with dyslexia (Ferrer et al., 2023). Interestingly, typical readers showed more variation in grade 1 reading scores than those with dyslexia, while students with dyslexia showed more variation in reading progress between grades 1 and 5 than typical readers (Ferrer et al., 2023). A valuable follow-up analysis reported that receiving special reading services during elementary school was *not* positively associated with reading performance in adulthood (~75% of the dyslexia sample participated in interventions), which the authors interpreted as related to interventions that were not aligned with evidence-based best practice (details on curricula or related information were not reported; Ferrer, Toste, Shaywitz, Holahan, & Shaywitz, 2025).

The inclusion of contextual data related to reading experiences can enhance our understanding of trajectories, as illustrated in research with related populations. Maughan et al. (2009) included metrics of adolescent and early adult print exposure, formal education/training, out-of-school reading in adolescence, and workplace literacy demands, all of which showed some associations with spelling skills in adulthood. Snowling, Muter, and Carroll (2007) measured a wide range of factors, reporting that children with a family history of dyslexia and literacy impairments showed significantly more emotional symptoms and lower self-ratings of scholastic competence, as compared to unimpaired readers with family history of dyslexia and typical readers. Insights from these types of variables may help us better understand the impact of both formal programs and broader literacy experiences on reading development, as well as how reading performance can be evaluated relative to educational resources. These data are important not only because they offer context for reading progress, but also in recognition that many levels and factors interact (e.g., genes, brain networks, cognition, motivation, socio-emotional context, and educator skills and knowledge) during the dynamic process of reading acquisition (Bonte & Brem, 2024).

## CASE STUDY: PRACTICE–RESEARCH COLLABORATION

To complement research on reading trajectories for students with RD drawn from students enrolled in US public schools, we report analyses of students attending a private school tailored for struggling readers. Conducted as part of a research–practice collaboration, the Brain, Education, and Mind (BEAM) Lab (Christodoulou and Mesite) at the MGH Institute of Health Professions and Landmark School (LS) partnered to analyze student performance data for grades 2–8, representing Elementary and Middle School (EMS), and grades 9–12, representing High School (HS), spanning the fall of 2012 through 2019. The extant data were collected by LS staff as part of their standard of practice for enrolled students. LS is a tuition-based school serving students with dyslexia and other language-based learning disabilities (LBLD), the majority of whom experience RD. In Appendix A, we provide context for the LS community related to teacher training, literacy instruction, and participant demographics, which may be important for generalizability potential, especially considering students enrolled in other school settings with RD may not have comparable resources to those available at LS.

### Word Reading Trajectories

We report test scores for untimed real word reading and pseudoword reading for LS students. We focus on word-level skills because they serve as the main barrier for students with dyslexia, and the LS curriculum includes an emphasis on this area. We explored students’ developmental trajectories using growth scale values (GSVs) from the Woodcock Reading Mastery Test 3rd Edition (Woodcock, 2011). GSVs, derived from raw scores, describe the participants’ change over time on an equal interval scale. For example, in WRMT Form A, the Word Identification GSV score range is 337–599 (corresponding to raw scores of 0–46) and the Word Attack GSV score range is 421–547 (corresponding to raw scores of 0–26).

We estimated growth separately for real word (WRMT Word Identification) and pseudoword (WRMT Word Attack) reading among students in elementary and middle school (EMS) (*n* = 388) and HS (*n* = 432). Since students were assessed at different points throughout the school year, we modeled GSVs as a function of grade level, which was a continuous variable reflecting time within the academic year (e.g., 3.0 = start/September of 3rd grade, 3.5 = midyear of 3rd grade). A linear mixed-effects model was used, with grade level (fractional) as a fixed effect and a random intercept for students to account for repeated observations and the difference in number of observations across students (min = 2, max = 8).

**Fig. 1.**
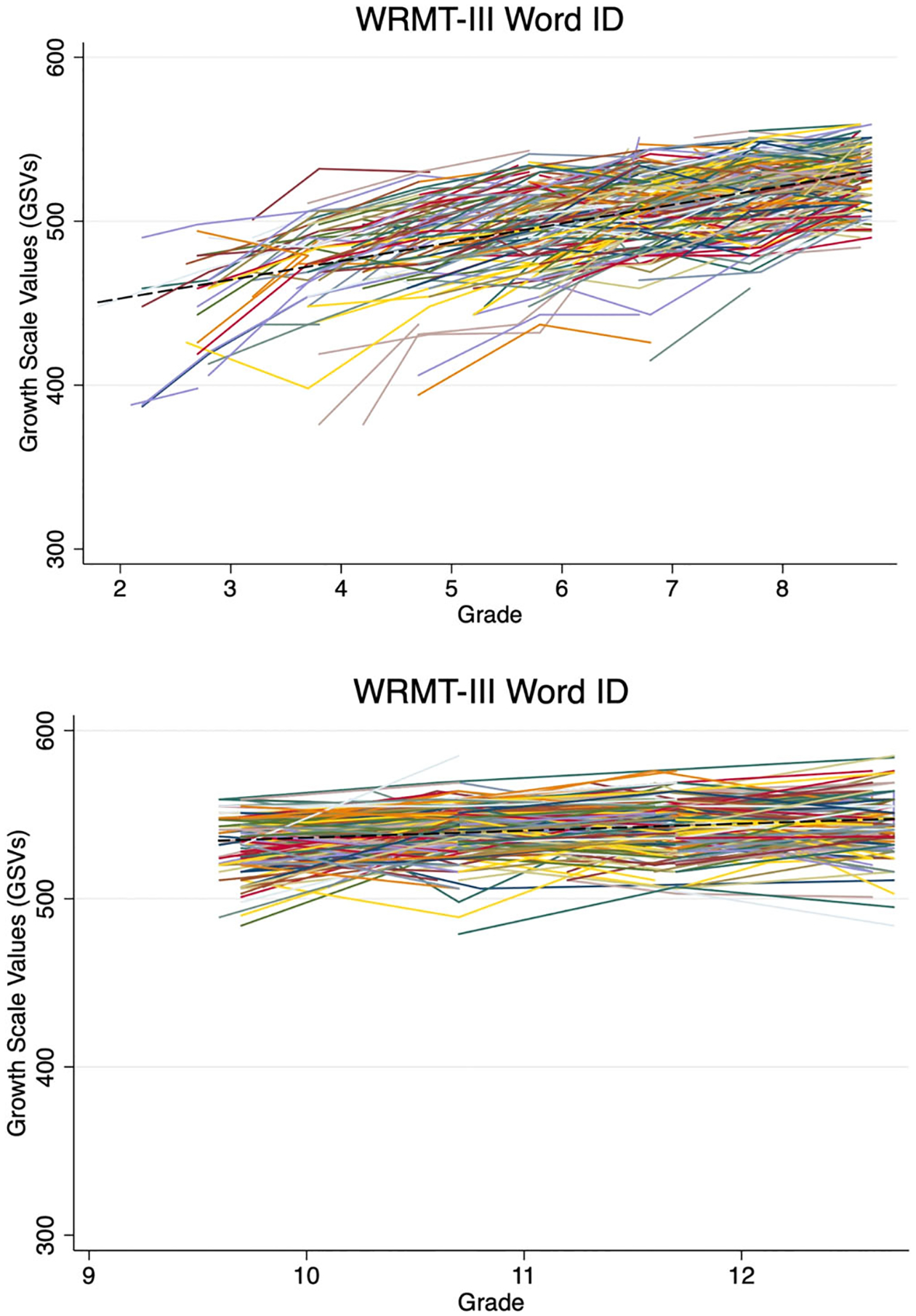
Untimed real word reading Growth Scale Values across grades 2–8 (EMS) and 9–12 (HS).

For real word reading abilities, EMS students gained an average of 12.8 GSVs per grade level on WRMT Word Identification, *b* = 12.82, SE = [0.31], *z* = [41.96], *p* < .001. During HS, students gained approximately 4.5 GSVs per year, on average, *b* = 4.54, *SE* = [0.32], *z* = [14.17], *p* < .001. Figure 1 demonstrates the predicted average growth trajectories (dashed line) as well as individual student score changes (solid lines).

### Untimed Pseudoword Reading

**Fig. 2.**
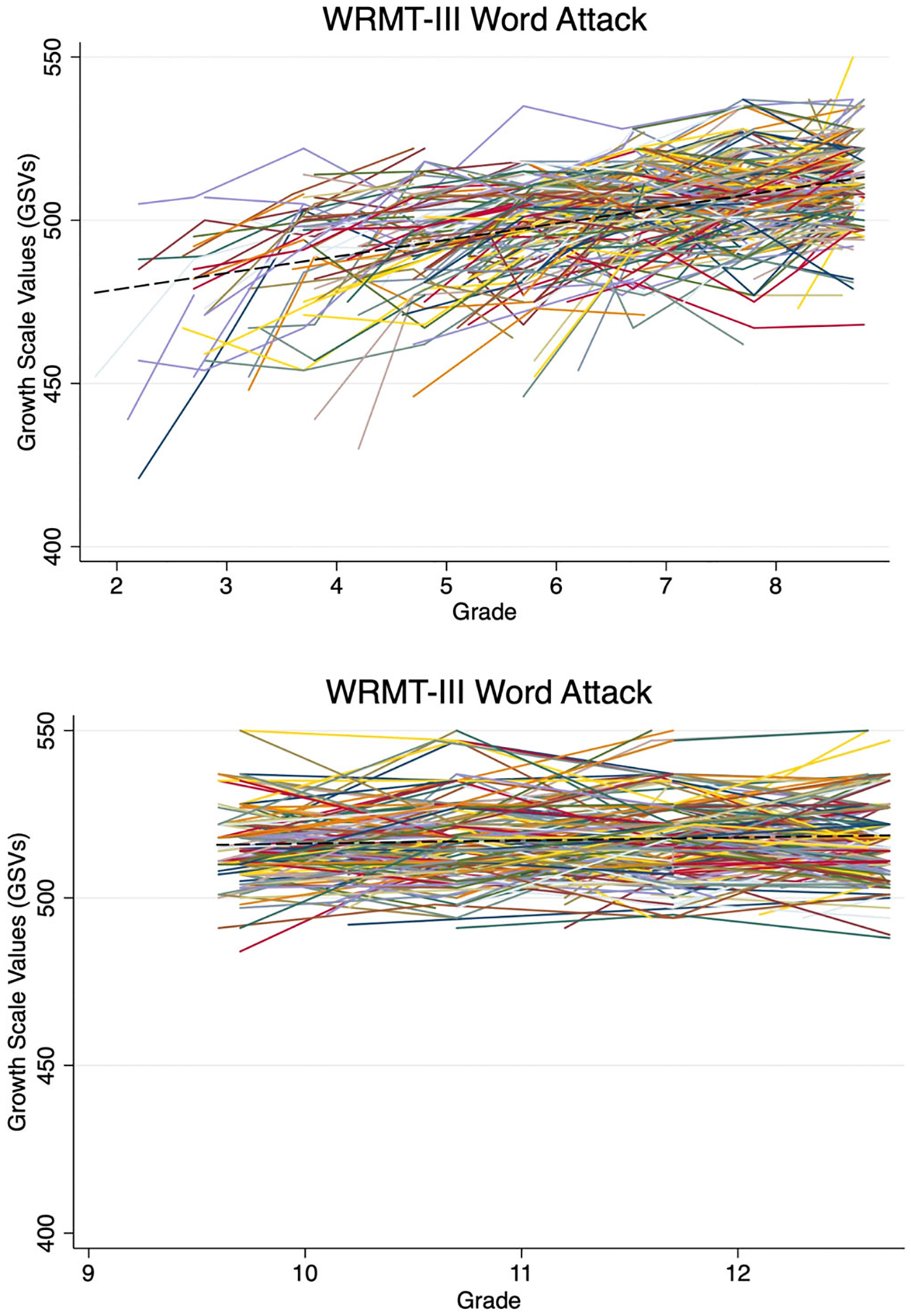
Untimed pseudoword reading Growth Scale Values across grades 2 to 8 (EMS) and 9 to 12 (HS).

For pseudoword reading abilities, EMS students gained an average of 5.7 GSVs per grade level on WRMT Word Attack, *b* = 5.73, *SE* = [0.22], *z* = [26.16], *p* < .001. For HS students, untimed pseudoword reading improved slightly across grades, with gains of one and a half GSVs per school year, *b* = 1.52, SE = [0.24], *z* = [6.36], *p* < .001. Figure 2 demonstrates the average growth trajectories (dashed line) as well as individual student score changes (solid lines).

### Contextualizing LBLD Trajectories

**Fig. 3.**
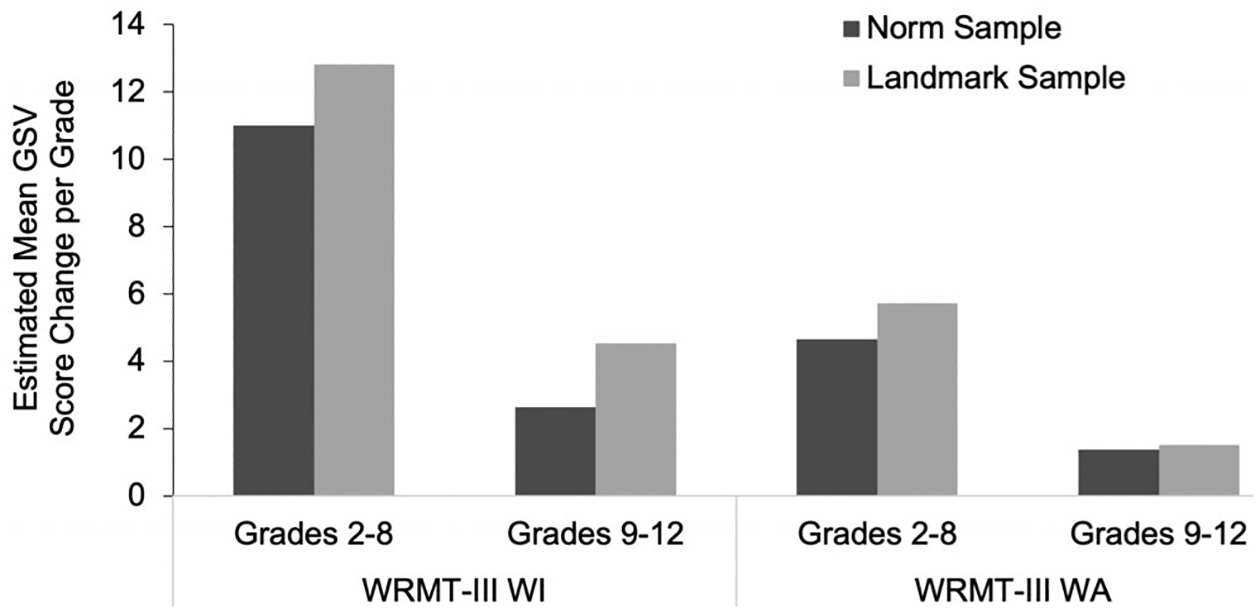
WRMT-III Growth Scale Values estimated change comparisons: Norm sample and Landmark School sample.

This case study offers an exploration of the reading trajectories for children with RDs attending a school tailored for this population. While the current dataset does not have a direct comparison to peers (e.g., students without RD or those with RD in public schools or other settings), evaluative comparison with GSVs based on the normative sample of the WRMT-III yields some helpful metrics. (See Appendix B for GSV calculations). The prototypical average reader increases, per school year, 11 GSVs from grades 2 to 8 and 2.6 GSVs from grades 9 to 12 for WRMT-III WI, on average. These estimates are compared to the LS sample: 12.8 GSVs per year for grades 2–8 and 4.5 GSVs per school year for grades 9–12 for WRMT-III WI, on average. On WRMT-III WA, the prototypical average reader gains 4.6 GSVs from grades 2 to 8 and 1.4 GSVs from grades 9 to 12, on average per school year. In comparison, the LS sample showed gains of 5.7 GSV points for EMS and 1.5 GSV points for HS. (See Figure 3.) Given prior findings reporting largely stable reading trajectories for individuals with RD that consistently trail their non-RD peers (Connors et al., 2025; Shaywitz et al., 1999), these findings offer an initial exploration of growth potential for students attending a school specialized in serving struggling readers compared to a grade-matched norm sample of prototypically average readers.

While we expect individual differences and nonlinear changes over time (e.g., word reading scores are expected to increase more in elementary school and less in HS; Shaywitz et al., 1999), this type of work can improve our knowledge about the range and variability of reading changes over time for readers with RD, and be used to identify the most impactful levers of change to improve reading outcomes. We recognize that additional measures would be useful to enhance our understanding of reading progress over time and overcome any potential limitations of a single tool. We also acknowledge that statistical concerns such as regression to the mean and selection bias may complicate interpretations of reading trajectories; this emphasizes the critical need for research using carefully matched groups of students with RD and their peers. Future work including children with RDs across multiple educational settings (e.g., varieties of public schools, other specialized schools tailored to LBLD communities, private schools, and homeschool settings) will be valuable additions to this work, as will be studies exploring broader socio-emotional well-being.

## SKILL PROGRESSION FOLLOWING THE END OF INTERVENTION PROGRAMS

### Fade-out Effects

The immediate impact of reading interventions is often studied, but we bring attention to their potential long-term sequelae months to years after an intervention is completed. Studies focused on these extended timeframes have frequently identified a ‘fade-out effect’ referring to the diminishing persistence of intervention gains over time. In one broad meta-analysis of intervention effects over time, consistent evidence of fade-out effects was shown across a range of cognitive programs (including 80% language- or literacy-focused curricula) and socioemotional programs (Hart, Bailey, Luo, Sengupta, & Watts, 2024). This work reports a conditional persistence rate of 45% at 6- to 12-month follow-up, and 18% at 1- to 2-year follow-up. The conditional persistence rate captures the component of a follow-up intervention effect on cognitive skills that is driven by posttest intervention effects on cognitive skills (i.e., how stable intervention impacts are at posttest and follow-up). Much of this research has focused broadly on early childhood interventions; studies explicitly examining long-term effects of reading interventions remain limited (Hart et al., 2024). One meta-analysis on long-term reading intervention outcomes (i.e., average of 11 months after participating in interventions) found that (1) overall effect sizes were reduced from posttest (*d*_w_ = 0.37) to follow-up (*d*_w_ = 0.22), on average, and (2) readers identified as at-risk, low, or disabled, in comparison to typical readers, show greater retention of intervention effects at follow-up (Suggate, 2016). Further identifying the features of successful intervention ecosystems spanning participants, curricula, and educators will be essential for designing scalable, sustainable models that produce lasting reading gains across readers and settings.

Several factors contribute to the limited follow-up after intervention studies, as described next based on Watts, Bailey, and Li (2019). Conducting long-term intervention studies is often cost-prohibitive, as funding is typically allocated to shorter implementation periods (e.g., typically up to 5 years for US grant funding mechanisms). Control groups may receive high-quality instruction during follow-up periods, complicating comparisons and reducing the availability of neutral benchmarks. Participants may not stay in the same location, making follow-up testing an additional challenge. Longer follow-up durations after reading interventions with students with RD can improve our understanding of how to sustain the benefits of interventions over time. This issue becomes even more significant when examining the impact of unexpected disruptions to formal education, which can amplify existing reading vulnerabilities.

## SUSPENDED FORMAL SCHOOLING

### Unplanned School Pauses

Unplanned disruptions to formal education, such as those caused by natural disasters, civil conflicts, or global health crises, underscore the importance of accessing reading instruction. The duration of school interruptions is difficult to anticipate in these contexts, as societal reactions evolve in response to challenges from these unexpected events and their consequences. These disruptions can affect access to education for students, the availability of school staff, learning resources, and the safety, health, and well-being of community members.

### COVID-19

Students enrolled in formal schooling during the interruptions from the COVID-19 pandemic (2020–2023) faced widespread disruptions. Many experienced the loss of loved ones, shifts in employment dynamics, constrained access to resources, and a transition to virtual social interactions. These factors contributed to heightened mental health needs and strained educational systems, particularly for special education services (Dvorsky et al., 2023).

Data from the National Assessment of Educational Progress (NAEP, 2008) showed that gaps were amplified during the COVID-19 school closure period (2020–2022) with the most vulnerable readers experiencing the greatest setbacks. For example, students performing at the 10th percentile for reading dropped by 10 scaled score points, while peers at the 90th percentile dropped by only two points, from 2020 to 2022 on NAEP measures. Likewise, students with disabilities declined from 187 to 180, while their peers shifted from 225 to 220 during this period (possible scores range from 0 to 500). In addition, students enrolled in lower grades (measured across primary and secondary school) experienced greater negative impact on reading outcomes (Amplify, 2022).

### Planned School Pauses

Planned breaks in formal schooling, such as summer vacations, introduce predictable interruptions in academic learning. In the northeastern United States, for example, students typically finish the school year in late June and return in early September. Summer vacations typically last 8–12 weeks, although their dates and duration vary by region and school type (e.g., public vs. private schools). The duration of summer breaks is related to the duration of the academic year, which is determined at the state level; the most common minimum number of school days is about 180 days (DeSilver, 2023).

Summer vacation can be a time of slowed reading growth for many students, including those with RDs. School-based summer reading programs can offer extended school-year services for students in special education to mitigate expected summer reading regression and to capitalize on opportunities for reading progress (Christodoulou, Azor, & Marks, 2023; Reed, Aloe, Reeger, & Folsom, 2019; Reed, Cook, & Aloe, 2020). Informed educators can better serve vulnerable readers during the summer by understanding reading trajectories specific to students with RD and the impact of summer literacy programming. Most research on reading trajectories centers on in-school periods, often over-looking the potential for growth during summer months. Extended breaks may be important to leverage for accelerating reading progress for vulnerable students through literacy programming.

### Summer Slide

The academic setbacks associated with summer vacation are often referred to as “summer slide.” Despite widespread colloquial recognition, research on summer outcomes lags relative to interest and relevance. We next describe selected findings relevant to summer reading outcomes from large datasets spanning students across the United States. However, we note that these large-scale studies include broad populations, not specific to RD, and future research is warranted to offer more tailored insights specific to RD.

Gershenson and Hayes (2017) used ECLS-K, a nationally representative dataset of children in kindergarten during 1998–1999. They compared reading gains during the summer between kindergarten and grade one for students with (*n* = 50) versus without (*n* = 1,250) IEPs. They report that students with IEPs made significantly (*p* < .05) larger summer reading gains than mainstream peers, reported as about 15% of a test score standard deviation if there were to be an additional 10 days of summer vacation. These gains may be associated with their higher reported enrollment in summer school. Analysis did not include type of disability, however, or details on activities during summer school to differentiate outcomes further.

Cooc and Quinn (2022) used data from ECLS-K:2011 to evaluate summer outcomes relative to school-year progress from kindergarten to grade two. They report widening gaps in reading during the school year between students with learning disability (*n* = 350) versus no disability at all (*n* = 13,430) across K-grade 2, and no significant differences in rates during the summer after K or grade 1. The researchers reported an overall lack of consistent findings for summer activity enrollment for students with disabilities.

Atteberry and McEachin (2021) used the Northwest Evaluation Association’s (NWEA) Measures of Academic Progress (MAP) Growth assessments to examine variability in summer learning outcomes relative to school-year performance among students in grades 1 through 8 (*n* = 17,955,222; not differentiated by RD status). They report a wide degree of variability across students for summer reading outcomes that are not well predicted by socioeconomic status, race/ethnicity, prior achievement, home variables, or summer activities, thus highlighting that summer reading outcomes are not dependent on a single factor or even a combination of factors often studied. However, 52% of students lost ground in *consecutive* summers (across grades 1–5); over 5 years, the accumulation of these summer lags for the prototypical student in this group is estimated to amount to 39% of total school-year gains.

Johnson and Barker (2023) analyzed summer reading outcomes between students who had ever received special education services or not (undifferentiated by disability type) followed from kindergarten to fourth grade. They used the NWEA MAP Growth assessments dataset with over 4,000 students. They also report that students who end up with access to special education services enter school at kindergarten with significantly lower reading-related scores than their peers. The achievement gap grew between K and 4th grade driven by greater decreases in summer reading scores for students receiving special education services, despite selected academic years of faster growth in reading by the group of children receiving special education services. Thus, the authors posit that accumulated summer lag appears to drive most of the achievement gap for students receiving special education services in this dataset.

Given the interest and impact of reading outcomes during the summer, we next address common questions that arise.

#### To what extent is a summer slowdown in reading progress a concern?

A slowdown in reading progress during summer breaks is a concern to the extent that it exacerbates existing achievement gaps or impedes vulnerable students from maintaining or advancing their skills. While quantifying summer reading outcomes provides useful averages, the emphasis should be on individual differences and heterogeneity, as broad averages may obscure the unique needs of specific students or subgroups. Evidence regarding the accumulation of summer setbacks to the same students over time emphasizes the importance of identification and intervention practices during the summer (Atteberry & McEachin 2021).

#### How does standard score change relate to raw score change?

To maintain the same standard score over time for reading performance, raw scores generally increase because of experiential expectations. Meaningfully interpreting raw score change is limited by the use of alternate forms across time points and when item-level data are unavailable. Notably, standard scores using grade-based norms are time-locked to the academic calendar, while those using age-based norms are tied to a child’s age. Developmental scale scores (i.e., GSVs) offer an alternative scoring scale overcoming many limitations by providing a stable comparison framework over time, independent of grade or age norms.

#### Does summer skill regression reflect true learning ‘loss’?

Terms like “learning loss” suggest attrition or deletion of skills, but current research does not provide the item-level data needed to explore this interpretation. To evaluate whether specific skills deteriorate, future studies should examine how performance on individual test items changes over time. Current findings rely on aggregated data, which obscures whether declines reflect true loss of skills or shifts in performance patterns. Additionally, it remains unclear which skills or knowledge are most vulnerable during episodes of reduced reading exposure (i.e., summer breaks). We posit, however, that score decreases during the summer reflect a slowing of growth for most students, and potential waning of access to prior (pre-consolidated) knowledge for vulnerable students.

## CONCLUSION

Improving reading outcomes for students with RD requires a multifaceted approach that extends beyond the content of interventions to consider the critical role of timing and contextual influences. This special issue addresses potential entry points for improving intervention outcomes via important content-based approaches and themes, including through consideration of spelling and complex word instruction (Compton et al., this issue; Steacy et al., this issue), oral narrative comprehension and production instruction (Gillam et al., this issue), strategies for inattention (Roberts et al., this issue), and modifying instructional elements (Clemens et al., this issue). Attention to students themselves was addressed via considerations of students’ oral language challenges (Snowling & Hulme, this issue), nonmainstream American English (Gatlin-Nash & Kim, this issue), reader subtype-based instruction (Schechter et al., this issue), machine learning insights (Ahmed et al., this issue), and intervention-based brain plasticity (Yeatman et al., this issue).

In addition to these important ‘what’ and ‘who’ considerations, we added a focus here on contextualizing the ‘when’ to improve how we understand progress in the broader circumstances of a reader’s interaction with their environment. Understanding learning trajectories provides a foundation for setting baseline expectations for progress, while addressing fade-out effects highlights the importance of sustaining gains achieved through continued supports and instruction. Periods of suspended formal schooling—whether planned, such as summer vacations, or unplanned, as during the COVID-19 pandemic—underscore the vulnerability of reading progress and the need for strategic supports during these times. Relatedly, the capacity to measure reading scores over time requires consideration of measures and score types, as some measures allow for longitudinal interpretation more readily (such as those with GSV scores).

Additional considerations for how we interpret students’ score changes (or lack thereof) are addressed in this issue by Francis, who focuses on the use of standardized tests for intervention research; by Tipton and Patton-Terry, who address how to best design studies when treatment effect heterogeneity is likely; by Wanzek and Change on the impact of intervention intensity; and by Leung et al. in distinguishing between non-replication and non-generalization.

In reviewing temporal contexts, we revisit opportunities for continued progress in addressing the needs of students with RD. The reliance on broad categorizations in large-scale datasets (e.g., special education status, IEP designation) limits the specificity of research findings, pointing to a need for studies that focus on specific subgroups, including students with RD. We emphasize the importance of tailoring interventions to individual needs while considering broader temporal contexts, which provide baseline expectations for conditions that may help or hinder progress over time. Specifying the period when interventions are implemented (e.g., school year vs. summer) and detailing additional literacy experiences (e.g., tutoring, special education services, home literacy experiences, and special education services) can help further inform factors impacting reading progress. Ultimately, advancing our understanding of when and how to implement reading support will better equip educators, researchers, and policymakers to promote enduring literacy gains for all learners, particularly those most vulnerable.

## Data Availability

The data that support the findings of this study are available from Landmark School. Restrictions apply to the availability of these data, which were used under license for this study. Data are available from the author(s) with the permission of Landmark School.

## APPENDIX A: LANDMARK SCHOOL COMMUNITY INFORMATION

### LANDMARK SCHOOL: PRIVATE SPECIAL EDUCATION SCHOOL

Landmark School belongs to the Massachusetts Association of 766 Approved Private Schools (MAAPS), which is an association of private special education schools in MA serving students with special needs based on the state’s special education law, Chapter 766.

### TEACHER TRAINING

Teachers at Landmark School who provide direct literacy instruction are required to have the following qualifications: Bachelor’s Degree, Ed.M. in Special Education, and Licensure in Moderate Disabilities PreK-8 or 5–12 from the State of Massachusetts. New educators are enrolled by Landmark School to Boston University Wheelock College of Education and Human Development to earn this degree and are supported in their licensure process. Complementing their studies, Landmark School provides intensive in-service professional development through a multiyear approach, including instructional coaching and robust mentorship.

### LITERACY INSTRUCTION

Students at Landmark School receive reading instruction provided in 1:1 and small-group settings. The 1:1 sessions (“Language Arts Tutorial”) are provided to students in grades 2–12 for a range of 40–80 min per day. The focus of each Tutorial is tailored to individual student literacy needs. A common principle of instruction is using a speech-to-print format to map phonology to orthography. The range of skills addressed in the Tutorial includes: phonemic awareness, phonics, oral reading fluency, vocabulary, comprehension, written expression, and spelling. Landmark uses a diagnostic and prescriptive approach to all literacy instruction; teachers identify literacy needs and remediate them prescriptively using linguistically controlled approaches.

Reading small-group instruction (1:6–8 teacher to student ratio) focuses on reading fluency, comprehension, vocabulary, and grammar. Here, teachers develop students’ metalinguistic knowledge to help move students to independence and provide an opportunity for students to step outside the processes of reading and reflect on their own strategy choices to increase reading efficacy. Instruction is grounded in a diagnostic/prescriptive approach that is tailored to student needs across grades.

### PARTICIPANT DEMOGRAPHICS

#### Race/ethnicity

Landmark School students, in general, are represented across racial and ethnic identities as follows: 85.7% White, 5.4% Black or African American, 5.4% two or more races, 4.1% Asian or Asian/Pacific Islander, 2.4% Hispanic/Latino, 1.5% American Indian or Alaska Native. In the current sample, the majority of participants (43%) did not provide theirs.

#### Occupational Prestige

Across EMS and HS students in this study, 85–88% had at least one parent with an occupational prestige score of 7 or higher on a 9-point scale, indicating a high prestige job (e.g., nurse, CEO, professor, doctor).

#### Sex

Across all participants (EMS and HS), about two-thirds of the students were male (64% male, 36% female).

### ADDENDUM TO METHODS

#### Model Description

We tested a random slopes model, but it did not significantly improve model fit, so we retained the random intercept model for parsimony. We also tested a quadratic term for grade level, which slightly improved model fit, but not substantially, and it reduced interpretability. Therefore, we retained the linear model.

## APPENDIX B: GROWTH SCALE VALUE CALCULATIONS FOR THE WRMT-III NORM SAMPLE

GSV score changes are next described using Form A grade-based norms for the prototypical average reader. The prototypical average reader is defined here as one whose GSV/raw score corresponds to a standard score at the population mean of 100 using fall, winter, and spring norms in each grade. For some time points, no GSV/raw score corresponds to a standard score of 100; where GSV/raw scores were only available corresponding to standard scores immediately above and below 100 (e.g., 98 and 102), we calculated the average GSV using these two values). The prototypical average reader GSV score range for WRMT WI is 464–541 from the fall of grade 2 to the spring of grade 8, and 542.5–553 from the fall of grade 9 to the spring of grade 12. For WRMT-III WA, the prototypical average reader GSV points increased from 484 to 516.5 from grades 2 to 8, and from 516.5 to 522 across grades 9 to 12.
